# Elevated MMP-8 levels, inversely associated with BMI, predict mortality in mechanically ventilated patients: an observational multicenter study

**DOI:** 10.1186/s13054-023-04579-3

**Published:** 2023-07-18

**Authors:** Hang Ruan, Shu-sheng Li, Qin Zhang, Xiao Ran

**Affiliations:** 1 Department of Critical-Care Medicine, Tongji Hospital, Tongji Medical College, Huazhong University of Science and Technology, 1095# Jiefang Ave, Wuhan, 430030 China; 2 Department of Emergency Medicine, Tongji Hospital, Tongji Medical College, Huazhong University of Science and Technology, Wuhan, China; 3 Department of Anesthesiology, Hubei Key Laboratory of Geriatric Anesthesia and Perioperative Brain Health, and Wuhan Clinical Research Center for Geriatric Anesthesia, Tongji Hospital, Tongji Medical College, Huazhong University of Science and Technology, 1095# Jiefang Ave, Wuhan, 430030 China

**Keywords:** Mechanical ventilation, Body mass index, MMP-8, Observational study, Machine learning

## Abstract

**Background:**

The present study aimed to investigate the correlation between weight status and mortality in mechanically ventilated patients and explore the potential mediators.

**Methods:**

Three medical centers encompassing 3301 critically ill patients receiving mechanical ventilation were assembled for retrospective analysis to compare mortality across various weight categories of patients using machine learning algorithms. Bioinformatics analysis identified genes exhibiting differential expression among distinct weight categories. A prospective study was then conducted on a distinct cohort of 50 healthy individuals and 193 other mechanically ventilated patients. The expression levels of the genes identified through bioinformatics analysis were quantified through enzyme-linked immunosorbent assay (ELISA).

**Results:**

The retrospective analysis revealed that overweight individuals had a lower mortality rate than underweight individuals, and body mass index (BMI) was an independent protective factor. Bioinformatics analysis identified matrix metalloproteinase 8 (MMP-8) as a differentially expressed gene between overweight and underweight populations. The results of further prospective studies showed that overweight patients had significantly lower MMP-8 levels than underweight patients ((3.717 (2.628, 4.191) vs. 2.763 (1.923, 3.753), ng/ml, *P* = 0.002). High MMP-8 levels were associated with increased mortality risk (OR = 4.249, *P* = 0.005), indicating that elevated level of MMP-8 predicts the mortality risk of underweight patients receiving mechanical ventilation.

**Conclusions:**

This study provides evidence for a protective effect of obesity in mechanically ventilated patients and highlights the potential role of MMP-8 level as a biomarker for predicting mortality risk in this population.

**Supplementary Information:**

The online version contains supplementary material available at 10.1186/s13054-023-04579-3.

## Background

Obesity augments the susceptibility to an array of illnesses, such as hypertension, hyperlipidemia, type 2 diabetes, coronary heart disease, stroke, and certain cancers, and is linked to amplified mortality and morbidity across diverse populations [1]. However, recent studies have suggested that mildly obese has a protective effect in specific clinical settings [2]. In some populations with chronic diseases, overweight or mildly obese patients exhibit a lowered mortality rate, i.e., the phenomenon is termed the obesity paradox [3–5]. Whether an obesity paradox exists in critically ill patients undergoing mechanical ventilation is yet controversial [6]. Furthermore, racial and ethnic disparities are noted in the obesity paradox [7]. Hence, additional studies are required to comprehend the association between weight status and mortality in diverse mechanically ventilated populations of different ethnicities.

Mechanical ventilation is a widely used respiratory support technique in intensive care medicine to assist or replace breathing in patients with respiratory failure. The repetitive opening and closing of alveoli during mechanical ventilation can cause shear stress and consequent mechanical damage, leading to the collapse of alveolar septa or deflection of fluid-filled alveoli, thereby resulting in uneven inflation within adjacent alveoli [8]. This uneven intraalveolar inflation can lead to uneven oxygen concentrations within the alveoli and locally high oxygen concentrations, which can lead to oxidative stress [7, 9–11]. Oxidative stress can cause damage to cellular components, impairing cell function and survival, and initiate pathological processes, such as lung damage and alveolar edema, which have an adverse impact on the effectiveness of mechanical ventilation [12, 13].

In the present study, we aimed to investigate the correlation between weight status and mortality in mechanically ventilated patients and explore the potential role of the expression of matrix metalloproteinase-8 (MMP-8), the reactive oxygen species (ROS)-related gene, in the obesity paradox among East Asian populations receiving mechanical ventilation. Herein, we conducted a retrospective analysis of clinical data from 3301 mechanically ventilated patients in three medical centres to examine the correlation between patient body mass index (BMI) and hospital mortality and validate the existence of the obesity paradox in the East Asian population using machine learning algorithms. Next, we obtained data from the gene expression omnibus (GEO) database for bioinformatic analysis and identified the oxidative stress-related gene matrix metalloproteinase-8 (MMP-8) with differential expression between different weight groups. Finally, we conducted a prospective study in a defined cohort of 243 subjects, using enzyme-linked immunosorbent assay (ELISA) to measure plasma MMP-8 levels to validate the differential expression of MMP-8 in patients with different body weights and investigate the correlation between MMP-8 and mortality. This study provided evidence for the protective effect of overweight or mildly obese in mechanically ventilated patients and highlighted the potential role of MMP-8 expression as a biomarker for predicting mortality risk in this population.

## Methods

### Study design and data collection

**Fig. 1:**
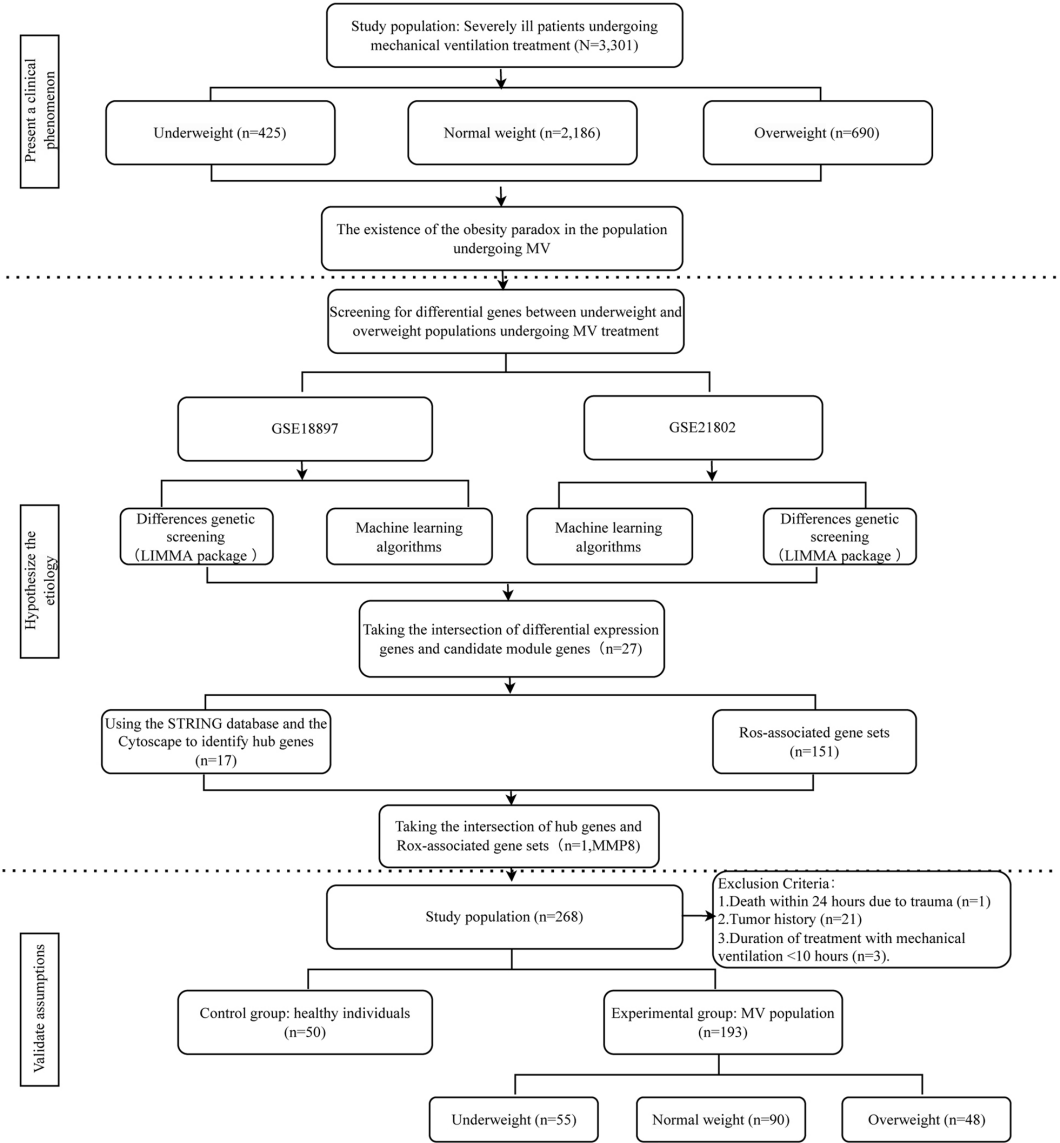
Flowchart of study design

This study was designed using both retrospective and prospective approaches, and clinical data were collected from three medical centres located in Wuhan, China: Tongji Hospital Sino-French New City Branch in Caidian District, Tongji Hospital Qiaokou Branch in Hankou District, and Tongji Hospital Optics Valley Branch in Wuhan East Lake High-Tech Development Zone. We conducted a retrospective study by collecting data from a total of 3301 critically ill patients who underwent mechanical ventilation over the past 5 years and analysed the correlation between their BMI and in-hospital mortality. The validation cohort was obtained from a prospective study conducted between October 2014 and September 2018 [14]. Within this study, patients were categorized based on their utilization of mechanical ventilation, and exclusion criteria were applied to obtain a final cohort comprising 50 healthy adults and 193 patients who had undergone mechanical ventilation. The study design is depicted in Fig. 1.

### Inclusion, exclusion, and subgroup criteria

Patients who were mechanically ventilated for at least 10 h were included in this study. Our collection of mechanically ventilated patients included patients treated with invasive mechanical ventilation, non-invasive mechanical ventilation, and high-flow nasal cannula. The following groups were excluded: (1) individuals < 18-years-old; (2) patients with acute cardiovascular and cerebrovascular events that resulted in death within 24 h; (3) those with severe trauma that resulted in death within 24 h. Additionally, the prospective cohort did not include patients with solid tumours. In this study, we collected a predominantly Chinese population, and the grouping criteria for BMI referred to Chinese standards: underweight: BMI < 18.5 kg/m^2^; normal range: 18.5 ≤ BMI < 24 kg/m^2^; overweight: BMI ≥ 24 kg/m^2^. The age grouping criteria pertain to the definition of older people’s age in developing countries and are categorized into two groups: those < 60-years-old and those ≥ 60-years-old.

### Data collection

The data were obtained from electronic medical records and encompassed various parameters, such as age, gender, BMI, arterial partial pressure of oxygen (PaO_2_), partial pressure of carbon dioxide, (PCO_2_); liver function indicators, kidney function indicators, coagulation function, sequential organ failure assessment (SOFA) score, and age-adjusted Charlson comorbidity index (aCCI). In-hospital mortality was the primary clinical outcome. Plasma samples were collected from a prospective cohort within 4 h of receiving mechanical ventilation. The locally weighted regression scatter plot smoothing (LOWESS) was utilized for preliminary exploration of the relationship between BMI and in-hospital mortality rate.

### Regression discontinuity design (RDD)

Herein, we employed restrictive cubic spline (RCS) analyses to evaluate the nonlinear correlation between BMI and mortality rate. We utilized RDD to evaluate the causal correlation between BMI and mortality rate. RDD analysis is similar to a randomized experiment [15]. It uses the sudden change point of the independent variable to explore the changes in the dependent variable, thereby inferring the causal correlation between the independent and dependent variables [16]. We utilized five machine learning models, including GaussianNB, LogisticRegression, DecisionTreeClassifier, RandomForestClassifier, and GradientBoostingClassifier, to obtain the optimal risk prediction model for predicting mortality risk in mechanically ventilated patients by comparing the area under the receiver operating characteristic (ROC) curve and model performance. The RDD with BMI was considered the grouping variable, and individual mortality risk was the outcome variable.

### Bioinformatics analysis

Bioinformatic analysis was performed to identify DEGs (differentially expressed genes) between overweight and underweight populations. To procure the dataset comprising individuals of varying weight statuses, we retrieved the GSE18897 dataset from the GEO database, consisting of 20 underweight and 20 overweight patients [17]. Similarly, we retrieved the mechanical ventilation dataset to obtain the GSE21802 dataset, which comprised four healthy individuals and six mechanically ventilated groups [18]. The source of the specimen was whole blood collected on the first day of admission. The gene expression profiles were extracted using the “GEOquery package” and the “limma package,” followed by the normalization of raw data [19, 20]. The intersection of the two gene sets was obtained to identify the DEGs during mechanical ventilation in patients with varying weight categories.

### Identifying differentially expressed genes

DEGs were identified based on the following criteria: |Log_2_Fold-Change|> 1 and *P* < 0.05. The variance threshold method in machine learning was applied to screen the feature genes. This method is a feature selection technique that relies on the variance of a feature. It operates on the principle of eliminating features with a variance below a specified threshold, as these features might have a low impact on the prediction of the target variable. Typically, features with high variance exhibit strong predictive capabilities for the target variable. Diversified approaches can be employed to compute feature variance, and one popular method is the use of median absolute deviation (MAD), a robust variance estimation technique that remains unaffected by outliers. The MAD value was calculated for each gene, and the median MAD value × 1.4826 was employed as the threshold for variance screening[21]. This process filtered out the genes with high variance and identified the feature genes. Finally, the top 20 genes based on their characteristic value were visualized using a heat map.

To obtain the co-expressed genes, we intersected the set of differential genes with the feature genes identified by machine learning algorithms. The protein–protein interactions (PPI) were established using the online database STRING (string-db.org) with medium confidence (0.4) [22]. To confirm the hub genes using topological analysis methods of the degree algorithm, we utilized the CytoHubba plugin, which is a plugin of Cytoscape 3.8.0 (University of California, San Diego, CA, USA) [23]. Next, we employed Gene Ontology (GO) enrichment analysis and Kyoto Encyclopedia of Genes and Genomes (KEGG) pathway enrichment analysis to demonstrate the molecular function and critical pathways linked to the intersection genes using the package “org.Hs.eg.db” and “clusterProfiler” [24, 25].

Next, to identify hub genes associated with oxidative stress, we downloaded oxidative stress-related genes from the “regulation of reactive oxygen species metabolic process” gene set in the MSigDB database (http://www.gsea-msigdb.org/gsea/msigdb/index.jsp). To investigate specific target genes related to the regulation of the ROS pathway, we used the intersection of hub genes and oxidative stress-related genes. Mechanically ventilated populations were frequently accompanied by various underlying diseases. We attempted to analyse the expression of MMP-8 in various diseases, as the origin of a critical illness often determines the risk of mortality [26]. Therefore, we analysed DEGs expression levels in 15 non-neoplastic (renal failure, liver failure, coronary heart disease, chronic heart failure, infective endocarditis, stroke, idiopathic pulmonary fibrosis, asthma, pneumonia, sepsis, Crohn’s disease, ulcerative colitis, primary biliary cholangitis, primary sclerosing cholangitis, chronic obstructive pulmonary disease) and 20 neoplastic diseases to investigate whether differences in underlying diseases have an impact on DEGs expression levels (Additional file 1: Fig. S1). The non-neoplastic sample expression profile was retrieved from the Gene Expression Omnibus (GEO) database and analysed using the GEO2R online tool (http://www.ncbi.nlm.nih.gov/geo/geo2r). The tumor sample expression profile were sourced from The Cancer Genome Atlas (TCGA) database and analysed with Sangerbox [27].

### Plasma sample collection

Within 4 h of admission, the whole blood samples of mechanically ventilated patients admitted to the intensive care unit (ICU) were collected for MMP-8 estimation. The blood samples were placed in tubes containing EDTA and a mixture of protease inhibitors (Sigma-Aldrich, MO, USA). The plasma was obtained by centrifugation of the blood at 3000 g, 4 °C for 15 min. The remaining blood samples were clarified by centrifugation and stored at − 80 °C.

### ELISA

Plasma MMP-8 levels were measured using a commercially available ELISA kit (HM11140, Bio-swamp, Wuhan, China) according to the manufacturer’s instructions (http://web.bio-swamp.com/productSpecification/29188).

### Statistical analysis

We performed bioinformatics analysis using R software (version 4.1.0) and machine learning using anaconda3 software. Stata14.0 software was utilized for statistical analysis. To assess the associations between the variables under investigation, Spearman correlation and partial correlation analyses were performed. Continuous variables were expressed as mean ± standard deviation (SD) or median (lower quartile—upper quartile) based on their distribution. Categorical variables were presented as frequencies and percentages. For continuous variables, when the data met the normal distribution and satisfied the homogeneity of variance test, t-test was employed for two-group comparison, and one-way analysis of variance (ANOVA) was used for three-group comparison. When the data met the normal distribution but not the homogeneity of variance test, the Welch t-test was utilized for two-group comparison, and Welch one-way ANOVA was used for three-group comparison. When the data did not meet the normal distribution, Wilcoxon test was used for two-group comparison, and Kruskal–Wallis test was employed for three-group comparison. The chi-square test was utilized to compare the categorical variables between groups. Logistic regression analysis was utilized to evaluate the correlation between weight status and mortality, adjusted for age, sex, aCCI, and SOFA score. The significance level is set at two-tailed *P* < 0.05.

### Ethical considerations

This study was approved by the ethics committee of the hospital where the study was conducted (Wuhan, China; Approval No. TJ-IRB20230313). This study was conducted in line with the Helsinki Declaration of 1975. As the study was observational study in nature, informed consent was exempted.

## Results

### Demographic and clinical characteristics of the study population

As shown in Table 1, the retrospective cohort consisted of 3301 mechanically ventilated patients, with the majority being young individuals (51.4%) with a male predominance (67.5%). The median aCCI score was 2 (1–3), and the median SOFA score was 8 (7–10). The overall mortality rate was 17.15%. The collective median BMI was 21.36 (19.86–23.5) kg/m^2^, while the median BMI for survivors was 21.47 (19.98–23.72) kg/m^2^ and 20.73 (19.18–21.95) kg/m^2^ for non-survivors (*P* < 0.001). Additionally, no gender-based variations were observed between the group of surviving and deceased individuals. Moreover, no significant differences were detected in the inhaled oxygen concentration, white blood cell (WBC) count, and 24-h urine output (*P* > 0.05). Furthermore, we display the primary diagnosis for admission and the primary cause for mechanical ventilation in Additional file 2: Fig. S2.

**Table 1:** Clinical characteristics and laboratory tests of the derivation cohort

Characteristics	Overall	Survivor	Non-survivor	*P*-value
n	3301	2735	566	
Sex				0.163
Female	1074 (32.5%)	904 (27.4%)	170 (5.1%)	
Male	2227 (67.5%)	1831 (55.5%)	396 (12%)	
Age				< 0.001
< 60	1698 (51.4%)	1471 (44.6%)	227 (6.9%)	
≥ 60	1603 (48.6%)	1264 (38.3%)	339 (10.3%)	
aCCI	2 (1–3)	2 (1–3)	3 (1–4)	< 0.001
SOFA	8 (7–10)	8 (7–10)	9 (7–12)	< 0.001
MAP, mmHg	105 (93–117)	106 (95–118)	100 (86–114)	< 0.001
FIO_2_	61 (51–71)	61 (51–70)	60 (50–71)	0.511
PCO_2_, mmHg	43 (38–50)	44 (39–51)	41 (35–47)	< 0.001
PaO_2_, mmHg	91 (87–96)	91 (87–95.5)	89 (84–96)	< 0.001
WBC, 10^12^/L	7.43 (5.19–10.94)	7.34 (5.25–10.655)	8.12 (4.73–12.17)	0.528
PLT, 10^9^/L	125 (67–193)	128 (71–195)	106.5 (40.25–184.75)	< 0.001
Lac, mmol/L	2.23 (1.68–3.04)	2.15 (1.65–2.91)	2.59 (1.9125–3.56)	< 0.001
APTT, S	36.7 (33.6–40.1)	36.5 (33.6–39.6)	37.95 (33.7–42.5)	< 0.001
Urine, mL	4105 (3924–4219)	4108 (3927–4219)	4098.5 (3904.5–4219.8)	0.704
ALT, U/L	15 (10–27)	15 (10–25)	16 (9.25–33.75)	0.003
AST, U/L	20 (15–34)	20 (15–32.5)	24 (16–48)	< 0.001
BUN, mmol/L	5.4 (3.92–7.5)	5.31 (3.9–7.4)	5.89 (4.1–8.2675)	< 0.001
Cr, µmol/L	65 (46–92)	63 (45–89.84)	74.5 (48–106)	< 0.001
BMI, kg/m^2^	21.36 (19.86–23.5)	21.47 (19.98–23.72)	20.73 (19.18–21.95)	< 0.001
Co-morbidities				
Congestive Heart Failure	2542 (77.01%)	2084 (63.13%)	448 (13.57%)	0.183
Renal failure	968 (29.32%)	763 (23.11%)	205 (6.21%)	< 0.001
Hepatic failure	164 (4.97%)	117 (3.54%)	47 (1.42%)	< 0.001
Tumor	745 (22.84%)	604 (18.30%)	141 (4.27)	0.143
Sepsis	3126 (94.70%)	2584(78.28%)	542 (16.42%)	0.216
ARDS	2845 (86.19%)	2354 (71.31)	491 (14.87)	0.670

### BMI is an independent protective factor against in-hospital mortality

Logistic regression-based univariate and multivariate analyses, as well as, variance inflation factor (VIF) test determined BMI is an independent protective factor. In the univariate analysis, BMI showed a significant protective effect with a P-value < 0.001. In the multivariate analysis, after adjusting for other potential confounding factors, BMI remained a significant protective factor with an adjusted odds ratio of 0.898 [95% confidence interval (CI): 0.873–0.925) (Table 2). Additionally, the VIF values for all variables were < 10, indicating no presence of multicollinearity (Additional file 3: Table S1). Therefore, our findings suggested a protective role of BMI against in-hospital mortality.

**Table 2:** Risk and protective factors for mechanically ventilated patients

Characteristics	Total(N)	Univariate analysis		Multivariate analysis	
		Odds Ratio (95% CI)	*P*-value	Odds Ratio (95% CI)	*P*-value
Sex	3301				
Female	1074	Reference			
Male	2227	1.150 (0.945–1.400)	0.163		
Age	3301				
< 60	1698	Reference		Reference	
≥ 60	1603	1.738 (1.446–2.089)	** < 0.001**	1.600 (1.220–2.100)	**0.001**
aCCI	3301	1.200 (1.145–1.258)	** < 0.001**	1.038 (0.948–1.136)	0.423
SOFA	3301	1.151 (1.117–1.185)	** < 0.001**	1.076 (1.029–1.125)	**0.001**
MAP, mmHg	3301	0.983 (0.978–0.987)	** < 0.001**	0.995 (0.989–1.001)	0.095
FIO_2_	3301	0.997 (0.990–1.005)	0.517		
PCO_2_, mmHg	3301	0.957 (0.947–0.968)	** < 0.001**	0.968 (0.955–0.982)	** < 0.001**
PO_2_, mmHg	3301	0.996 (0.989–1.002)	0.164		
WBC, 10^12^/L	3301	1.005 (0.999–1.011)	0.119		
PLT, 10^9^/L	3301	0.998 (0.997–0.999)	** < 0.001**	1.001 (0.999–1.002)	0.333
Lac, mmol/L	3301	1.203 (1.140–1.270)	** < 0.001**	1.250 (1.138–1.374)	** < 0.001**
APTT, S	3301	1.024 (1.013–1.035)	** < 0.001**	1.002 (0.990–1.014)	0.762
Urine, mL	3283	1.000 (1.000–1.000)	0.433		
ALT, U/L	3301	1.001 (1.000–1.001)	**0.016**	1.000 (0.999–1.001)	0.691
AST, U/L	3301	1.000 (1.000–1.001)	**0.021**	0.999 (0.999–1.000)	**0.003**
BUN, mmol/L	3301	1.021 (1.008–1.035)	**0.002**	0.959 (0.933–0.985)	**0.002**
Cr, µmol/L	3301	1.001 (1.000–1.001)	**0.029**	1.001 (1.000–1.002)	0.073
BMI, kg/m^2^	3301	0.902 (0.876–0.928)	** < 0.001**	0.899 (0.873–0.925)	** < 0.001**
Congestive Heart Failure	2542	1.162 (0.932–1.450)	0.183		
Renal failure	968	1.468 (1.213–1.776)	** < 0.001**	1.090 (0.864–1.376)	0.466
Hepatic failure	164	2.026 (1.426–2.879)	** < 0.001**	1.454 (0.990–2.135)	0.056
Tumor	754	1.171 (0.948–1.445)	0.143		
Sepsis	3126	1.320 (0.849–2.050)	0.217		
ARDS	2845	1.060 (0.8121–1.382)	0.670		

Bold indicates statistically significant* P* values

### Correlation between weight status and in-hospital mortality

**Fig. 2:**
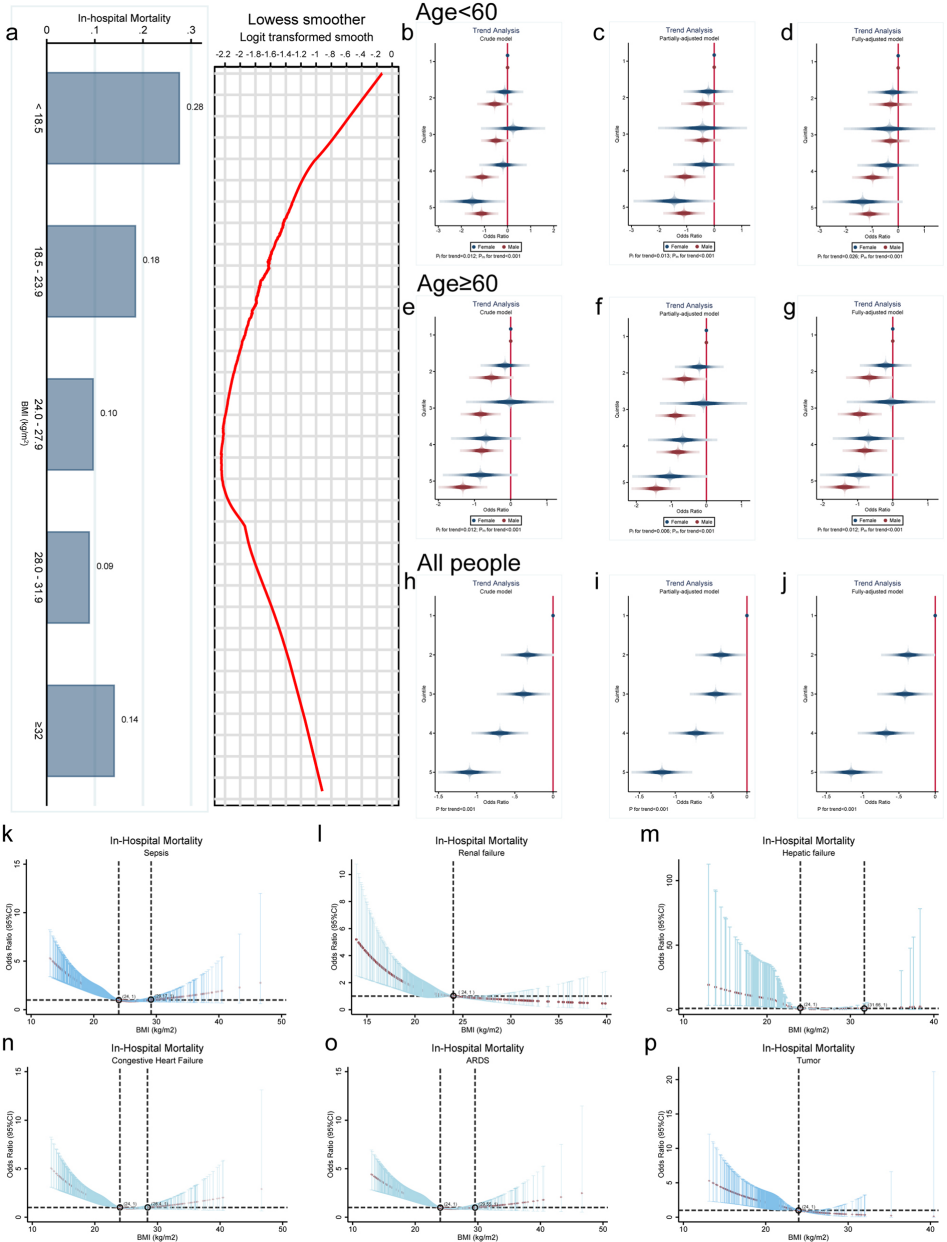
Exploratory analysis of the correlation between BMI levels and in-hospital mortality.** a** The LOWESS curve indicated a non-linear association between BMI and in-hospital mortality, approximating a U-shaped curve. **b**–**j** Quintiles categorized by BMI levels were utilized to calculate ORs for different age and gender groups. Males were represented by red, while females were represented by blue. The OR value for the confounders were adjusted asymptotically for crude, partial (SOFA, PaCO2, Lac, AST, BUN), and full (aCCI, SOFA, MAP, Fio2, PaCO2, PaO2, WBC, PLT, Lac, APTT, Urine, ALT, AST, BUN, Cr), as demonstrated in Table 2. Trend testing was conducted using* P*-values. The association between BMI and hospital mortality across Various Comorbidities. Note: BMI was divided into 5 groups based on quintiles (< 19.48, 19.49-20.76, 20.77-21.81, 21.82-24.15, >24.16, kg/m^2^).** k**–**p** Association of BMI with in-hospital mortality in subgroups with different co-morbidities (all* P* < 0.01)

The correlation between BMI and in-hospital mortality was initially investigated by utilizing the LOWESS curves. The analysis revealed a U-shaped association between BMI and in-hospital mortality, indicating that a slight increase in BMI led to a decrease in mortality (Fig. 2a). Furthermore, in order to gain a more in-depth understanding of the correlation between BMI and in-hospital mortality, we classified BMI into five groups based on quintiles. It was observed that the odds ratio (OR) of BMI decreased progressively with increasing quintile in any model, and the trend was statistically significant (all *P* for trend < 0.05, Fig. 2b–j). A non-linear correlation between BMI and in-hospital mortality was discovered in all subgroups of diseases after analysing various comorbidity subgroups (all *P* < 0.01, Fig. 2k–p).

**Fig. 3:**
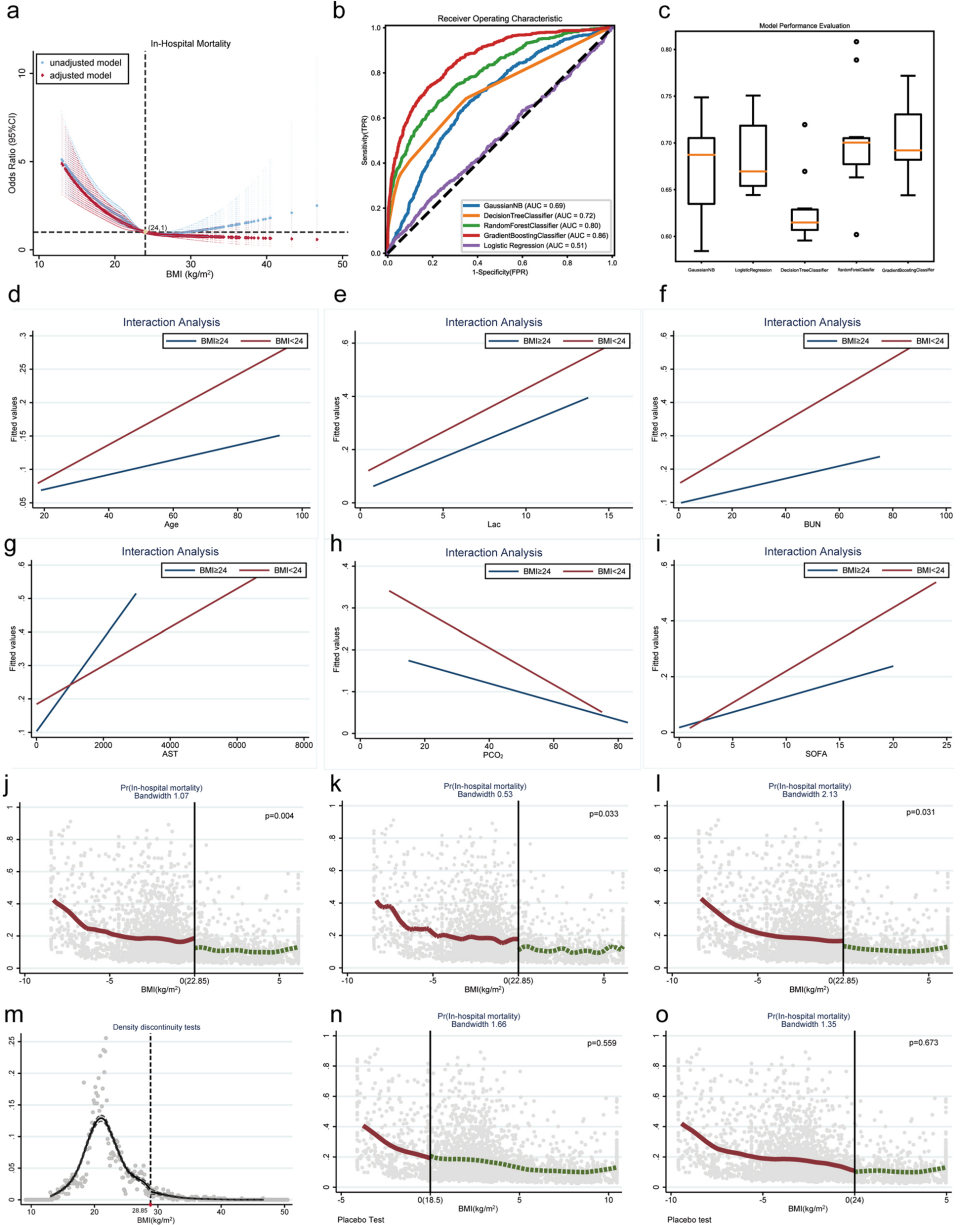
RDD analysis of the correlation between BMI and in-hospital mortality.** a** The RCS model indicates a nonlinear correlation between BMI and in-hospital mortality. In the unadjusted model, both the overall* p*-value and the nonlinear* P*-value were < 0.001. In the adjusted model, which accounted for confounding factors, such as age, SOFA, Paco2, Lac, AST, and BUN, the overall* P*-value was < 0.001 and the nonlinear* P*-value was = 0.022.** b** ROC curves for five machine learning models.** c** The model performance evaluation graph.** d**–**i** Interaction Analysis.** j** When BMI was 22.85 kg/m^2^, patients showed a significant reduction in the risk of in-hospital death.** k**, **l** Sensitivity analysis.** m** Continuity tests;** n**,** o** Placebo tests.

As shown in Fig. 3a, a nonlinear correlation was further detected between BMI and in-hospital mortality using RCS analysis (all *P* < 0.001). This correlation remained significant even after adjusting for potential confounding factors (all *P* < 0.05). Additionally, the different BMI ranges were compared with in-hospital mortality using the normal range of BMI as a reference. The observed trend is similar to that of RCS analysis (Additional file 3: Table S2). Of the five machine learning algorithm models, the Gradient Boost machine learning algorithm showed the highest accuracy (Fig. 3b, c**)**. The Gradient Boost machine learning algorithm was used to calculate the risk score for patients presenting in-hospital mortality. Interaction analysis showed the interaction of BMI with age (*P* for interaction < 0.001), Lac (*P* for interaction = 0.084), BUN (*P* for interaction = 0.004), AST (*P* for interaction = 0.014), PCO_2_ (*P* for interaction = 0.001) and SOFA (*P* for interaction < 0.001, Fig. 3d-i). Those covariates was controlled by conducting a RDD analysis.

RDD shows a significant reduction in mortality at BMI = 22.85 kg/m^2^ (*P* = 0.004, Fig. 3j), indicating a causal association between BMI and in-hospital mortality. In addition, sensitivity tests, continuity tests, placebo tests, and balance checks were conducted to demonstrate the robustness of the results (Fig. 3k–o, Additional file 3: Table S3). Therefore, these data indicate that the obesity paradox exist in East Asian populations receiving mechanical ventilation.

### MMP-8 was differentially expressed in underweight and overweight populations

**Fig. 4:**
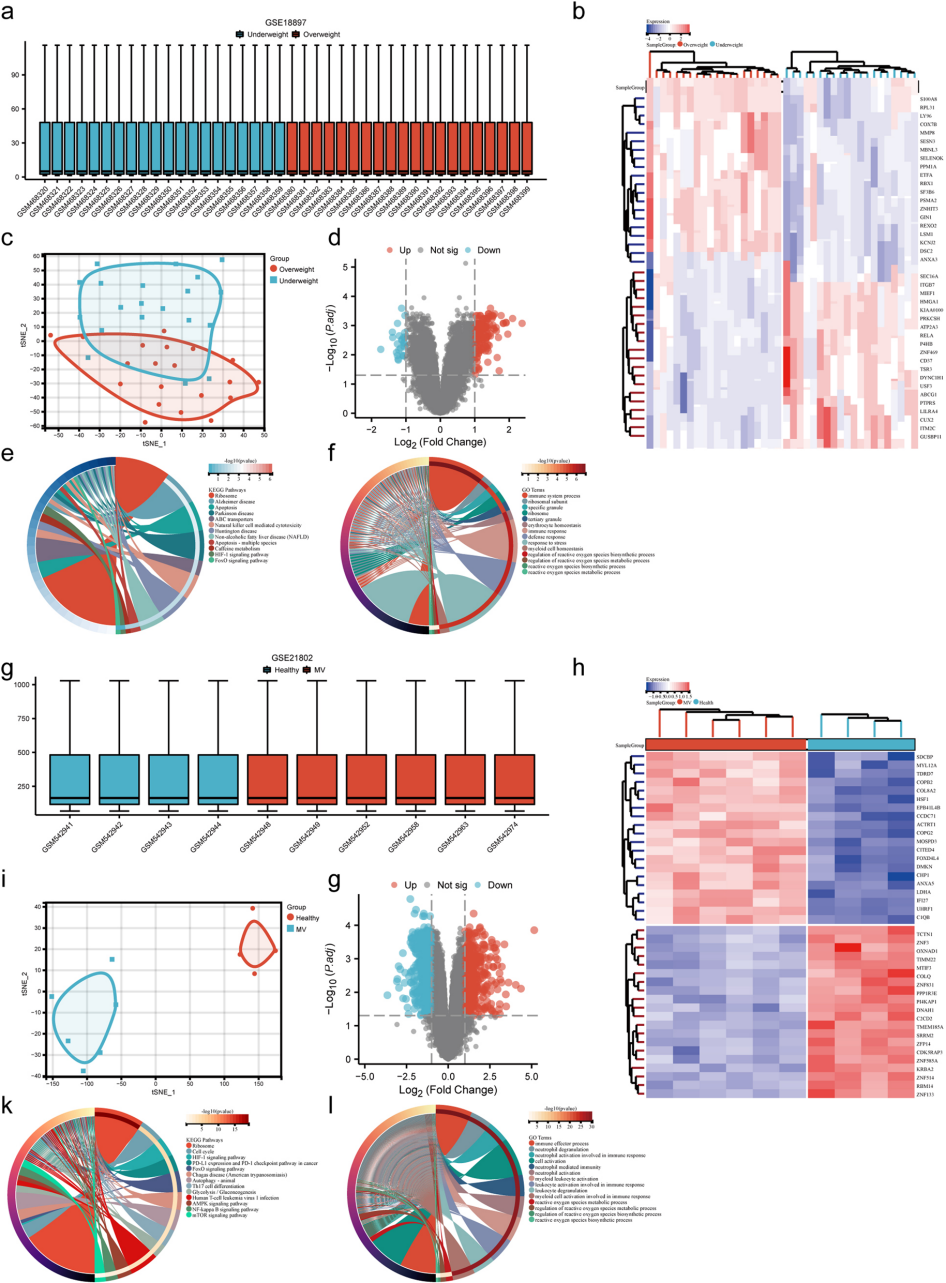
Results of the gene expression analysis for GSE18897 and GSE21802. **a** A box plot of normalized gene expression for the GSE18897 dataset. **b** A heatmap of DEGs. **c** A t-SNE plot. **d** A volcano plot of DEGs. **e** KEGG enrichment analysis of intersection genes. **f** GO enrichment analysis of intersected genes. **g** A box plot of normalized gene expression for the GSE21802 dataset. **h** A heatmap of DEGs. **i** A t-SNE plot. **j** A volcano plot of differentially expressed genes. **k** The KEGG enrichment analysis of intersection genes. **l** The GO enrichment analysis of intersected genes

A total of 173 DEGs were identified in the GSE18897 dataset using bioinformatics analysis, of which 147 were upregulated and 26 were downregulated (Fig. 4a–f). In the GSE21802 dataset, 1054 DEGs were identified, including 487 upregulated and 567 downregulated (Fig. 4g–l). Enrichment analysis was conducted separately for the differential genes in each dataset, and GO molecular function enrichment analysis revealed enrichment of reactive oxygen metabolism-related processes (Fig. 4e, k). KEGG pathway enrichment analysis indicated the presence of oxidative stress-related signaling pathways, such as the Foxo (Forkhead box O) and HIF1 (Hypoxia-inducible factor 1) signaling pathways, in both datasets (Fig. 4f, l).

**Fig. 5:**
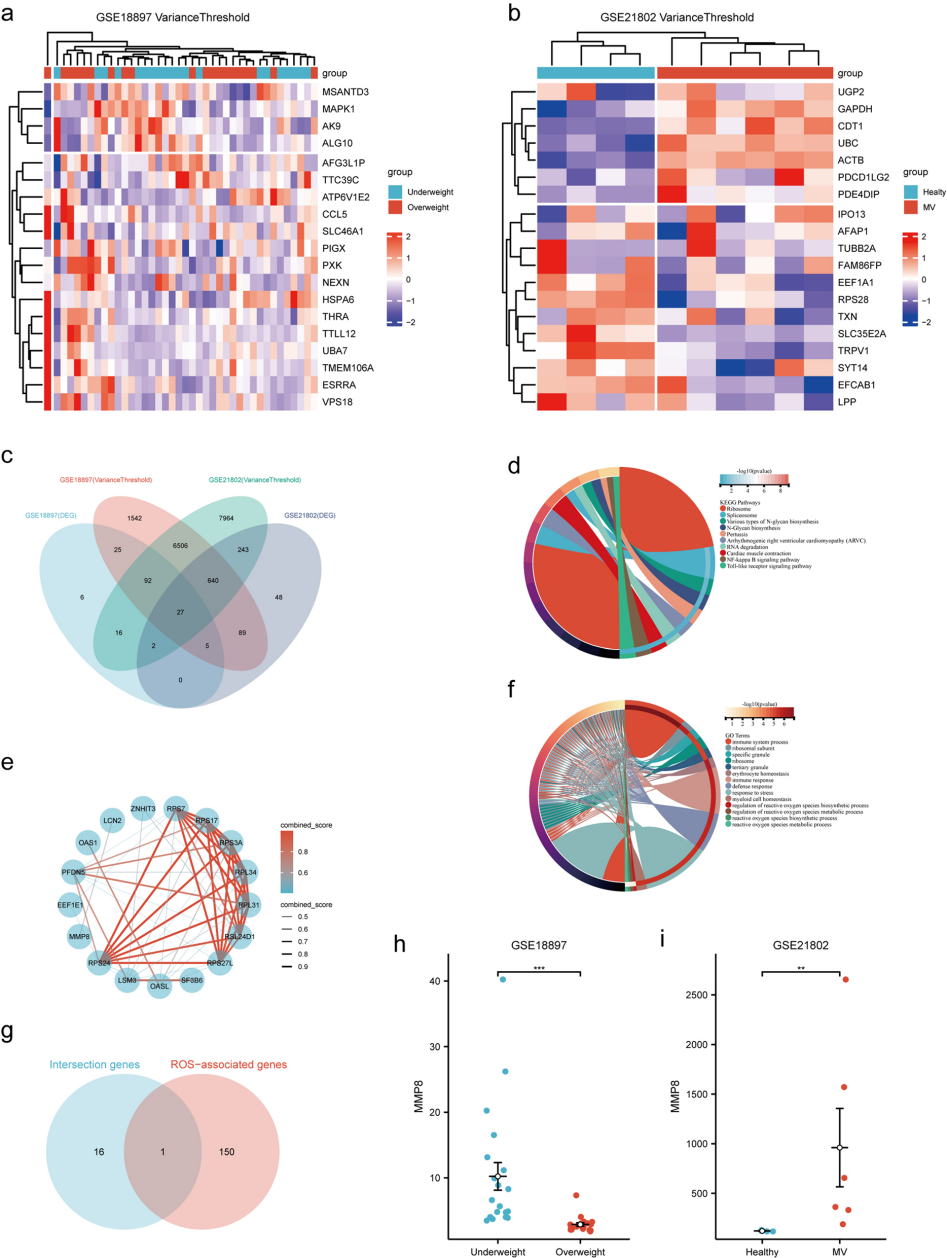
Identification and validation of oxidation stress-related hub genes. **a** Filtering features genes using variance thresholds for the GSE18897. **b** Filtering features genes using variance thresholds for the GSE21802. **c** A Venn diagram program was used to reflect the intersection between DEGs and feature genes. **d** GO enrichment analysis of intersection genes. **e** KEGG enrichment analysis of intersected genes. **f** PPI network of intersected genes. **g** Intersections of hub genes and oxidation stress-related gene set. **h-i** *MMP-8* mRNA expression level (*p* < 0.05). *Note* MV, mechanical ventilation

The variance threshold method identified 8926 and 15,490 signature genes in the GSE18897 and GSE21802 datasets, respectively (Fig. 5a, b). The intersection of the DEGs and signature gene datasets revealed 27 co-expressed genes (Fig. 5c). Further GO molecular function and KEGG pathway enrichment analysis of these 27 co-expressed genes suggested enrichment of oxidative stress-related signals (Fig. 5d, e). The PPI network and Cytoscape software identified 17 hub genes (Fig. 5f). To further identify the oxidative stress-related hub genes, an analysis was conducted between the hub genes and oxidative stress-related genes using a Venn diagram; the oxidative stress-related hub gene *MMP-8* was obtained (Fig. 5g). Significant differences were noted in *MMP-8* RNA levels in both datasets (GSE18897: 6.144 (4.034–11.662) *vs*. 2.657 (2.197–3.069), *P* < 0.001, Fig. 5h; GSE21802: 119.926 (116.764–126.732) *vs*. 508.967 (332.292–1570.505), *P* = 0.011, Fig. 5i**)**, indicating that MMP-8 may have differential expression in patients with varying weights while receiving mechanical ventilation.

After examining the levels of MMP-8 expression in non-oncological illnesses, noteworthy distinctions were discovered in severe asthma, pneumonia, and sepsis (|Log2Fold-Change|> 1 and *P* < 0.05). Subsequently, upon further scrutinizing the MMP-8 expression levels in both tumor and normal tissues, noteworthy variations were discovered across various tumor types, with particularly heightened expression evident in gastrointestinal tumor and lung cancer (Additional file 1: Fig. S1, Additional file 3: Table S4).

### Elevated MMP-8 levels predict the mortality risk of underweight patients receiving mechanical ventilation

To authenticate the findings of the bioinformatics analysis, we executed a prospective study comprising 50 healthy individuals and 193 mechanically ventilated patients. Demographics and clinical characteristics of the study population are shown in Table 3. In comparison to the healthy population, patients receiving mechanical ventilation exhibited a considerably higher concentration of plasma MMP-8 (2.702 (2.305–3.313) *vs.* 3.027(2.336–3.959) ng/ml, *P* = 0.030). Subsequently, we categorized all mechanically ventilated patients into three subgroups based upon their BMI levels: underweight (BMI < 18.5 kg/m2, n = 55), normal weight (BMI: 18.5–24 kg/m2, n = 90), and overweight (BMI > 24 kg/m2, n = 48). The demographic characteristics of the three groups, including age and gender, were not significantly different. Notably, plasma MMP-8 levels, as well as in-hospital mortality rates, varied significantly among the three groups of patients (all *P* < 0.05). Further analysis demonstrated that overweight patients had considerably lower MMP-8 levels than underweight patients ((3.717 (2.628- 4.191) vs. 2.763 (1.923- 3.753), ng/ml, *P* = 0.002). The mortality rates of patients in underweight, normal weight, and overweight groups were 13.6%, 15.6%, and 7.4%, respectively (Table 4). In contrast to the normal weight group, the obese group showed a prolonged mechanical ventilation duration (30 (24, 37) VS. 44.5 (26, 70), *P* < 0.001), albeit with a statistically significant reduction in ICU hospitalization time (123.5 (64–225) vs. 83 (56.5–149), *P* = 0.036). These findings together suggest that higher levels of MMP-8 indicate a higher risk of mortality for underweight patients undergoing mechanical ventilation.

**Table 3:** Mechanically ventilated validation cohort

Characteristics	Healthy (n = 50)	MV patients (n = 193, kg/m^2^)			*P*-value
		18.5–24 (n = 90)	≥ 24 (n = 48)	< 18.5 (n = 55)	
MMP-8, ng/ml	2.702 (2.305–3.313)	2.961 (2.447–3.611)	2.763 (1.923–3.753)	3.717 (2.628–4.191)	0.030*, 0.002^+^
Sex, female (n%)	30 (12.3%)	39 (16%)	17 (7%)	24 (9.9%)	0.019*, 0.619^+^
Age < 60, years (n%)	40 (16.5%)	61 (25.1%)	29 (11.9%)	34 (14%)	0.034*, 0.626^+^
BMI, kg/m^2^	21.95 (19.8–22.5)	22.95 (21.5–23.7)	24.8 (24.1–26.05)	17.9 (17.0–18.0)	0.193*, < 0.001^+^
aCCI	0 (0–2)	2 (1–3)	2 (1–4)	2 (1–4)	< 0.001*, 0.998^+^
SOFA	4 (3–5)	9 (8–11)	10 (8–11.5)	10 (8–12)	< 0.001*, 0.406^+^
MAP, mmHg	56 (51–83)	75 (60–98)	74 (64.5–90.5)	73 (61–82)	0.003*, 0.689^+^
FIO_2_	21 (21–21)	45 (37–54)	47 (40–50.5)	50 (41–60)	< 0.001*, 0.057^+^
PCO_2_, mmHg	42 (35–43)	37 (31–42)	37 (34–40.5)	39 (31–44)	0.007*, 0.448^+^
PaO_2_, mmHg	94 (89–98)	101.5 (78–116)	92 (77–119.5)	105 (88–131)	0.101*, 0.234^+^
WBC, 10^12^/L	8.88 (7.2–10.62)	15.865 (11.93–19.4)	14.88 (10.31–16.76)	15.1 (9.72–18.24)	< 0.001*, 0.259^+^
PLT, 10^9^/L	133.5 (123–144)	127 (83–164)	136 (66.5–159.1)	124.5 (63–169)	0.084*, 0.994^+^
Lac, mmol/L	0.73 (0.41–0.77)	5 (2.98–7.93)	4.925 (3.245–7.05)	4.22 (2.64–7.67)	< 0.001*, 0.593^+^
APTT, S	32.7 (31.5–37.8)	50.9 (41.2–60.6)	51.3 (42.85–64.2)	52.2 (41.9–64.2)	< 0.001*, 0.544^+^
Urine, mL	1303 (1203–1550)	1525 (1000–2150)	1660 (1187–2065)	1646.3 (945–2000)	0.090*, 0.548^+^
ALT, U/L	17.5 (12–41)	35 (14–84)	21.5 (14.5–46.5)	36 (14–159)	0.011*, 0.269^+^
AST, U/L	21 (16–29)	47 (23–111)	45 (23.5–76.5)	60 (25–261)	< 0.001*, 0.460^+^
BUN, mmol/L	4.39 (3.2–5.43)	10.55 (6.55–18)	11.25 (7.175–16)	9.15 (6.41–19.6)	< 0.001*, 0.890^+^
Cr, µmol/L	55.125 (48–73)	144 (67–271)	141 (84.5–272)	115 (75–237)	< 0.001*, 0.564^+^
Co-morbidities	–				
CHF	–	22 (11.40%)	7 (3.63%)	13 (6.74%)	0.378
Renal failure	–	37 (19.17%)	20 (10.36%)	20 (10.36%)	0.817
Hepatic failure	–	46 (23.83%)	22 (11.40%)	31 (16.06%)	0.566
Sepsis		77 (39.90%)	42 (21.76%)	43 (22.28%)	0.372
ARDS	–	68 (35.23%)	37 (19.17%)	44 (22.80%)	0.825

*: Comparisons between healthy and mechanically ventilated groups. + : Within MV subgroup comparisons. IHM, In-hospital mortality; CHF, Congestive Heart Failure; MV, Mechanical Ventilation

**Table 4:** Correlation of different BMI subgroups with clinical outcomes

Characteristics	MV patients (n = 193, kg/m^2^)			*P*-value
	18.5–24 (n = 90)	≥ 24 (n = 48)	< 18.5 (n = 55)	
*Clinical outcomes*				
In-Hospital Mortality, n (%)	38 (15.6%)	18 (7.4%)	33 (13.6%)	**0.044**
Length of ICU stay, hours	123.5 (64–225)	83 (56.5–149)	102 (58–209)	0.1009
MV time, hours	30 (24, 37)	44.5 (26, 70)	72 (35, 156)	** < 0.001**
*Co-morbidities*				
CHF	22 (11.40%)	7 (3.63%)	13 (6.74%)	0.378
Renal failure	37 (19.17%)	20 (10.36%)	20 (10.36%)	0.817
Hepatic failure	46 (23.83%)	22 (11.40%)	31 (16.06%)	0.566
Sepsis	77 (39.90%)	42 (21.76%)	43 (22.28%)	0.372
ARDS	68 (35.23%)	37 (19.17%)	44 (22.80%)	0.825

CHF, Congestive Heart Failure; MV, Mechanical Ventilation

Bold indicates statistically significant* P* values

### Associations of MMP-8 with different parameters

The heat map demonstrated that MMP-8 expression levels correlated with numerous laboratory indicators (Additional file 4: Fig. 3). In a comparison of subgroups by weight status, MMP-8 levels were significantly higher in the underweight group than in the normal weight and overweight groups (all *P* < 0.05). Spearman's correlation analysis further showed a significant negative correlation between MMP-8 and BMI (r = − 0.143, *P* < 0.05). The correlations retained their significance even after patriating out the covariation estimates (all *P* < 0.05).

**Fig. 6:**
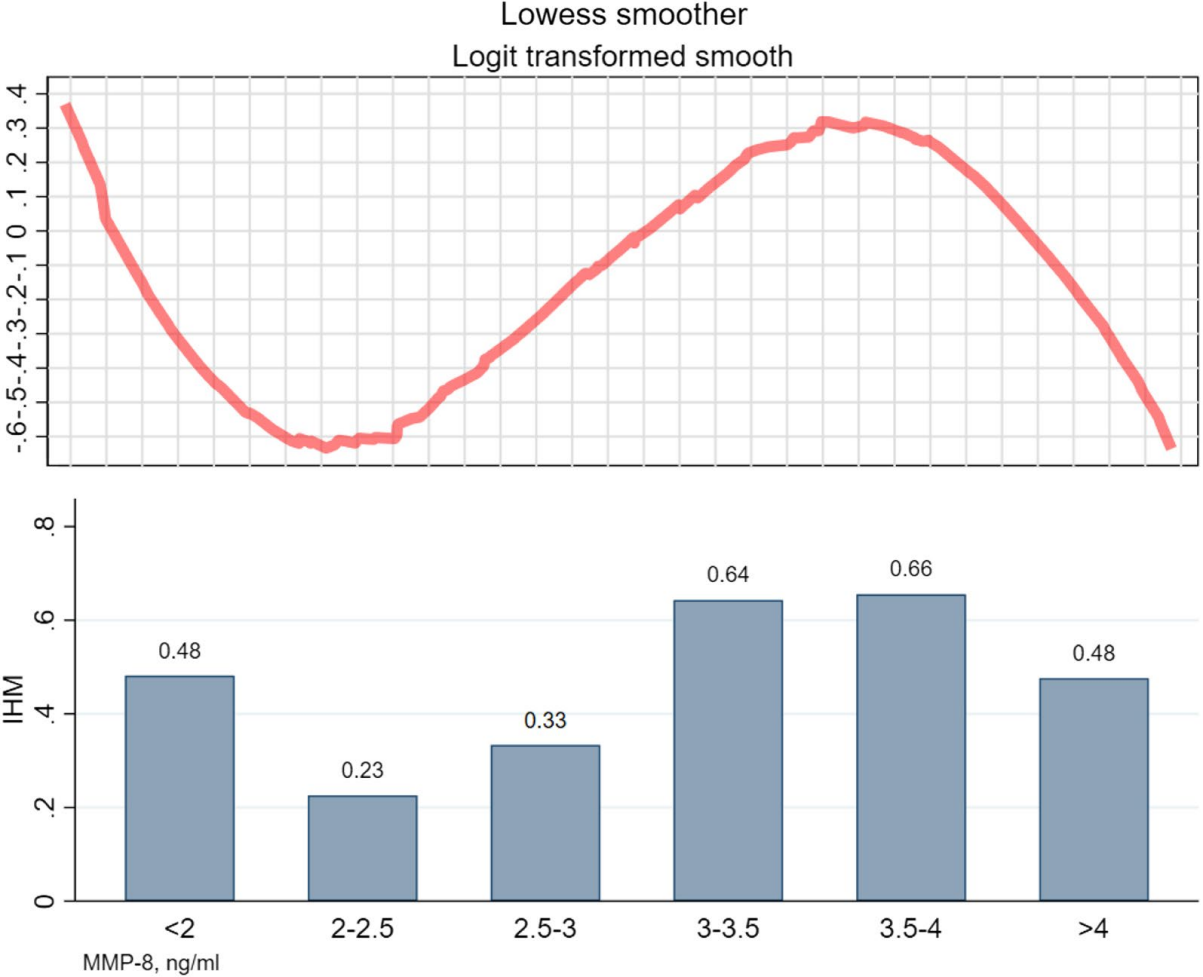
Correlation between MMP-8 levels and in-hospital mortality in mechanically ventilated patients. *Note* This is a combination of a Lewis smoothing curve and a histogram. IHM, in-hospital mortality

The dose–response correlation between patient MMP-8 expression levels and in-hospital mortality was examined using the LOWESS curve, which revealed an inverse S-shaped association between MMP-8 expression levels and ICU mortality. As illustrated in Fig. 6, patients were categorized into six groups based on MMP-8 concentration, and the mortality rates were computed for each group. The results of the LOWESS curve analysis were supported by the histograms. Furthermore, the S-shaped association was confirmed using a cubic term regression model (Additional file 3: Table S5). An S-shaped correlation was established between MMP-8 expression levels and in-hospital mortality, with two extreme points of (2.322, -0.929) and (3.867,0.903), respectively, having a trinomial coefficient (*P* < 0.001).

Due to an inverse “S” correlation between MMP-8 and in-hospital mortality, we conducted a segmented logistic regression analysis using the two vertices as cutoff points. The results revealed a significant association between high levels of MMP-8 and increased mortality risk. Specifically, MMP-8 levels (2.322–3.867 ng/mL) were associated with an odds ratio of 4.249 (*P* = 0.005) for mortality risk (Table 5). However, the results were not significant when the MMP was > 3.867 ng/mL or < 2.322 ng/mL (all *P* > 0.05), indicate that there is an increased risk of mortality in underweight patients undergoing mechanical ventilation with an increase in MMP-8 levels (OR (95% CI) = 4.249(1.546–11.675), *P* = 0.005). Therefore, MMP-8 level may serve as a potential predictive indicator of mortality risk in this population.

**Table 5:** Segmented logistic regression models

MMP-8, ng/ml	n	OR	(95% CI)	z	*P*-Value
< 2.322	46	0.069	0.003–1.422	− 1.73	0.083
2.322–3.867	94	4.249	1.546–11.675	2.80	0.005
> 3.867	53	0.136	0.010–1.891	− 1.94	0.138

Finally, we explore the value of MMP-8 as a molecular marker to predict in-hospital mortality. The study put forward a composite index that combined MMP-8 levels and relevant clinical parameters, including BMI, Lac, PAO2, SAO2, FIO2, and BUN. The composite index showed promising potential for diagnosis, outperforming the reference SOFA score in predicting in-hospital mortality (0.869 (0.816–0.922) vs. 0.600 (0.519–0.681), *P* < 0.001, Additional file 4: Fig. S3, Additional file 3: Table S6).

## Discussion

The obesity paradox is observed in East Asian populations receiving mechanical ventilation, with obesity emerging as an independent protective factor for mechanically ventilated patients based on the findings of our retrospective cohort study. Subsequently, we conducted bioinformatic analysis to identify potential mediators and eventually identified MMP-8 as a pivotal element in the emergence of this paradox in mechanically ventilated patients. We subsequently assessed the level of MMP-8 expression in various disease gene sets and found a significant increase in its expression level among the sepsis, severe asthma, and pneumonia groups as compared to healthy individuals. This finding suggests that hypoxia and infection are the principal factors underlying the rise in MMP-8. Given that critically ill patients requiring mechanical ventilation often exhibit a high incidence of hypoxia and infection, the elevated levels of MMP-8 observed in this cohort can likely be attributed to these common medical conditions.

To corroborate our findings, we carried out a prospective cohort study, which once again demonstrated a lower mortality rate in the overweight group when compared to the underweight group, confirming previous research highlighting the existence of the obesity paradox in critically ill patients [2]. Furthermore, we opted not to include tumor patients in our prospective cohort since MMP-8 expression levels vary considerably across different types of tumours. We also observed differential expression of MMP-8 in patients with varying weight statuses, indicating its potential as a biomarker for predicting clinical outcomes in this group. Currently, the underlying mechanisms of this paradox are inconclusive and can be attributed to various factors, including enhanced immune function, increased energy reserves, and reduced catabolic responses. Our study contributes new insights into the paradox from the perspective of oxidative stress.

Our study also found an inverted S-shaped association between MMP-8 and hospital mortality. Due to the lack of dose–response studies on MMP-8 levels in mechanically ventilated populations, we propose a potential mechanism for the inverse S-shaped association between MMP-8 and in-hospital mortality. High-serum MMP-8 levels have been reported to be associated with white blood cell counts in the literature[28]. Furthermore, existing studies suggest that elevated levels of MMP-8 in the serum are linked to systemic inflammation and adverse outcomes [29], indicating that the expression of MMP-8 in patients is related to the body's inflammatory response to external stimuli. Prior to the first nadir, the inflammatory response is inadequate, and as MMP-8 gradually rises, the inflammatory response strengthens, leading to reduced mortality (*P* = 0.083). When MMP-8 exceeds the threshold, a threshold effect occurs, indicating an excessive inflammatory response in the organism and causing mortality to rise rapidly with increasing MMP-8 levels (*P* = 0.005). Once the maximum point is surpassed, a saturation effect takes place, and the association between in-hospital mortality and MMP-8 is no longer statistically significant (*P* = 0.138).

MMP-8, known as collagenase-2 or neutrophil collagenase, belongs to the MMP family and expressed extensively within various cell types, including endothelial cells, vascular smooth muscle cells, neutrophils, and macrophages [30, 31]. Neutrophils predominantly store MMP-8 in gelatinase granules, which are released at the site of inflammation during inflammatory responses [32, 33]. Under normal physiological conditions, MMP-8 is stored as an inactive proenzyme and is biologically non-functional. However, in the presence of inflammation and ROS, MMP-8 undergoes activation through a cysteine conversion mechanism, ultimately converting from an inactive to an active form. Additionally, hypoxia enhances the process of neutrophil degranulation, leading to the transfer of multiple proteases to the extracellular compartment [34]. These phenomena might account for the rise in MMP-8 levels during mechanical ventilation.

The present study also discovered elevated levels of MMP-8 in lean patients, consistent with previous reports [35]. In a mouse model of blunt chest trauma, lean mice had significantly higher levels of MMP-8 in the first 6 h compared to obesity [36]. Additionally, a population-based study showed that plasma MMP-8 levels were lower in obese women than in lean women [35], consistent with our conclusion that MMP-8 expression differs across weight categories. Our study also demonstrated a correlation between MMP-8 and disease severity in mechanically ventilated patients, with higher MMP-8 levels associated with increased in-hospital mortality. It has been demonstrated in the colon cancer population that heightened serum MMP-8 levels are correlated with inflammatory responses and unfavorable outcomes, thus substantiating the notion [29]. Therefore, MMP-8 may serve as a potential biomarker for predicting in-hospital mortality in this patient population. Two potential reasons contribute to these findings: Firstly, mechanical ventilation is known to cause some degree of lung damage, and the level of MMP-8 is closely linked to lung tissue damage [37]. Secondly, increased levels of MMP-8 have been linked to poor prognosis, and clinical studies conducted on patients with severe COVID-19 lung disease revealed significantly higher levels of MMP-8 and MMP19 in the non-survival group compared to the survival group [38]. The underlying mechanism may be that the overexpression of MMP-8 increases lipid peroxidation and exacerbates lung injury [38].

This study has the following strengths. To begin with, it provides evidence for the protective effect of obesity in mechanically ventilated patients. This finding has clinical implications for the management of critically ill patients that may guide decision-making regarding nutritional support and weight management in this population. Moreover, this study highlights the potential role of MMP-8 expression as a biomarker for predicting mortality risk in mechanically ventilated patients. The identification of a biomarker accurately predicts that mortality risk can have significant implications for patient care, allowing early interventions and potentially improving outcomes. Finally, the study takes a multidisciplinary approach, integrating retrospective and prospective designs, bioinformatic analysis, and ELISA to gain a nuanced understanding of the underlying mechanisms and potential clinical implications of these findings. The multidisciplinary approach allows for comprehensive comprehension of the mechanisms underlying these findings and their potential clinical implications. Nevertheless, the present study has several limitations. Firstly, the population of critically ill patients receiving mechanical ventilation is highly heterogeneous, encompassing a range of diseases that cause severe respiratory failure, such as severe pneumonia, interstitial pneumonia, and chronic obstructive pulmonary disease. Moreover, different comorbidities, such as age, genetic susceptibility, and sources of infection, can complicate the level of MMP-8 expression. Although the analysis incorporated SOFA and aCCI scores to assess the states of various diseases, it is still challenging to eliminate infections as confounding factors. Secondly, due to the clinical nature of this study and the absence of in vitro cellular experiments, differences in MMP-8 mRNA and protein expression levels under cellular experiments were not analyzed. Thirdly, there are limitations in the BMI indicator, which only takes into account height and weight and cannot distinguish between muscle and fat. Also, BMI does not reflect fat distribution. Finally, our study, conducted from a clinical perspective, has revealed a correlation between MMP-8 and BMI, suggesting that MMP-8 may be a potential underlying cause of the obesity paradox. However, to further explore the causal relationship between MMP-8 and low BMI, we require the support of fundamental scientific methods. Thus, this will also be a key direction of our future research.

## Conclusions

This study provides new insights into the correlation between weight status and mortality rates in mechanically ventilated patients. Overweight and mild obesity is an independent protective factor, and MMP-8 is identified as a potential biomarker for predicting the outcomes in non-neoplastic condition. Further studies are required to validate the findings and explore the underlying mechanisms, which could have significant clinical implications and improve patient outcomes.

## Supplementary Information

**Additional file 1**. Differential expression of MMP-8 in diffrent diseases.

**Additional file 2**. The distribution of diseases among hospitalized patients.

**Additional file 3**. The relationship between MMP-8 expression levels and various clinical parameters.

**Additional file 4**. Appendix of Supplementary Table.

## Data Availability

The data and code used in this study could be obtained from the corresponding author upon reasonable request.

## Abbreviations

ALT: Alanine aminotransferase
APTT: Activated partial thromboplastin time
ARDS: Acute respiratory distress syndrome
AST: Aspartate aminotransferase
BLCA: Bladder urothelial carcinoma
BMI: Body mass index
BRCA: Breast invasive carcinoma
BUN: Blood urea nitrogen
CHOL: Cholangiocarcinoma
COAD: Colon adenocarcinoma
COADREAD: Colon adenocarcinoma/rectum adenocarcinoma esophageal carcinoma
DEGs: Differentially expressed genes
ELISA: Enzyme-linked immunosorbent assay
ESCA: Esophageal carcinoma
FIO2: Fraction of inspired oxygen
Foxo: Forkhead box O
GEO: Gene expression omnibus
GO: Gene ontology
HIF1: Hypoxia-inducible factor 1
HNSC: Head and Neck squamous cell carcinoma
IQR: Interquartile range
KEGG: Kyoto encyclopedia of genes and genomes
KICH: Kidney Chromophobe
KIDNEYPAN: Pan-kidney cohort (KICH + KIRC + KIRP)
KIRC: Kidney renal clear cell carcinoma
KIRP: Kidney renal papillary cell carcinoma
LIHC: Liver hepatocellular carcinoma
LUSC: Lung squamous cell carcinoma
LUAD: Lung adenocarcinoma
MAD: Median absolute deviation
MAP: Mean arterial pressure
MMP: Matrix metalloproteinase
MMP-8: Matrix metalloproteinase-8
MV: Mechanical ventilation
OCD: Obsessive–compulsive disorder
PaO2: Arterial partial pressure of oxygen
PLT: Platelet count
PPI: Protein–protein interactions
PRAD: Prostate adenocarcinoma
RDD: Regression discontinuity design
READ: Rectum adenocarcinoma
ROC curve: Receiver operating characteristic curve
RCS: Restrictive cubic spline
SD: Standard deviation
SOFA: Sequential organ failure assessment
STAD: Stomach adenocarcinoma
STES: Stomach and esophageal carcinoma
TCGA: The cancer genome atlas
THCA: Thyroid carcinoma
UCEC: Uterine corpus endometrial carcinoma
WBC: White blood cell count
