# Supplementary figures and images for: Elevated MMP-8 levels, inversely associated with BMI, predict mortality in mechanically ventilated patients: an observational multicenter study
Source: Crit Care. 2023 Jul 18;27:290. doi: 10.1186/s13054-023-04579-3 (PMC10355076; doi:10.1186/s13054-023-04579-3)

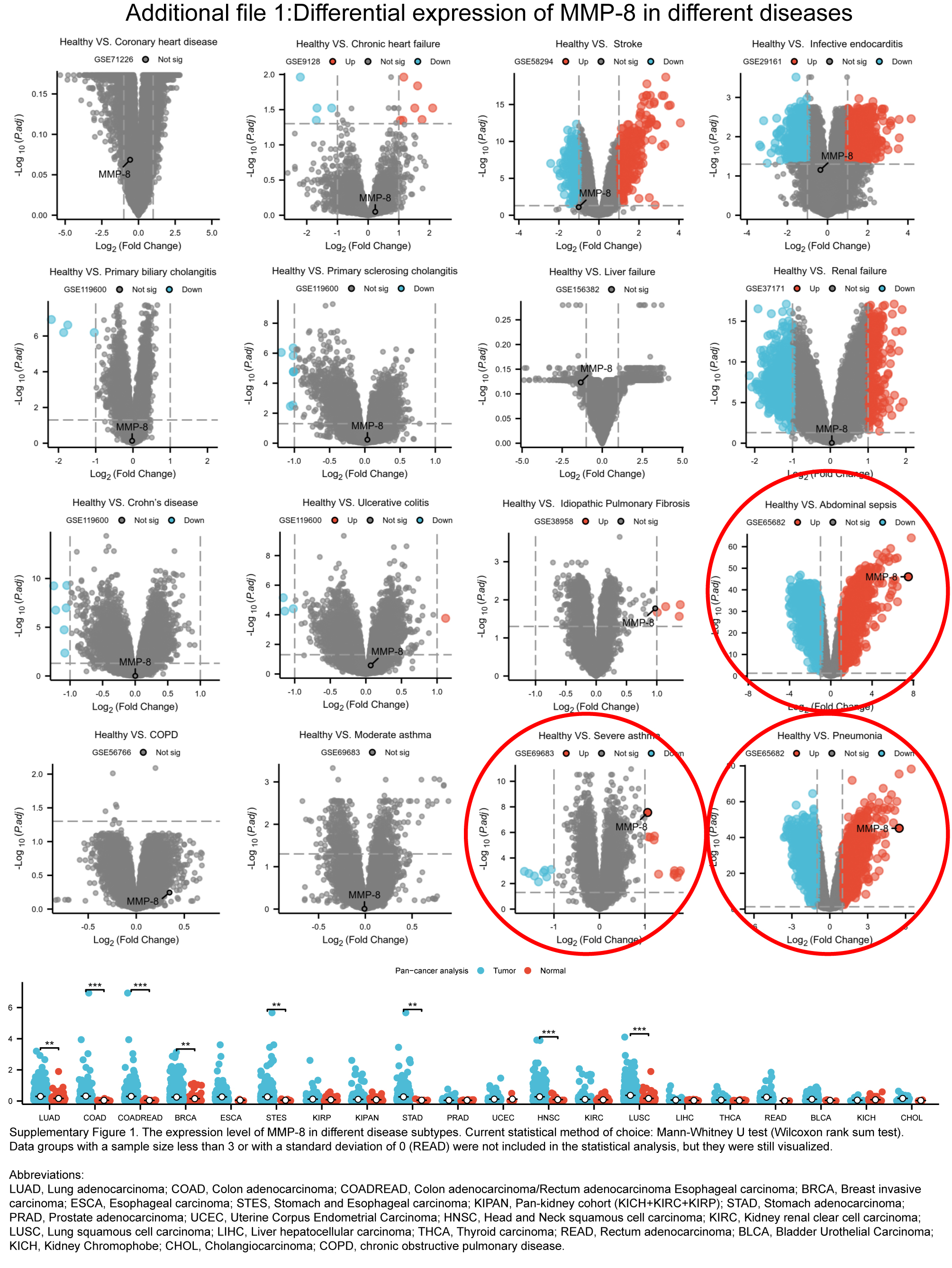

Supplement: Supplementary file 1 — Additional file 1. Differential expression of MMP-8 in diffrent diseases. [file 13054_2023_4579_MOESM1_ESM.jpg]

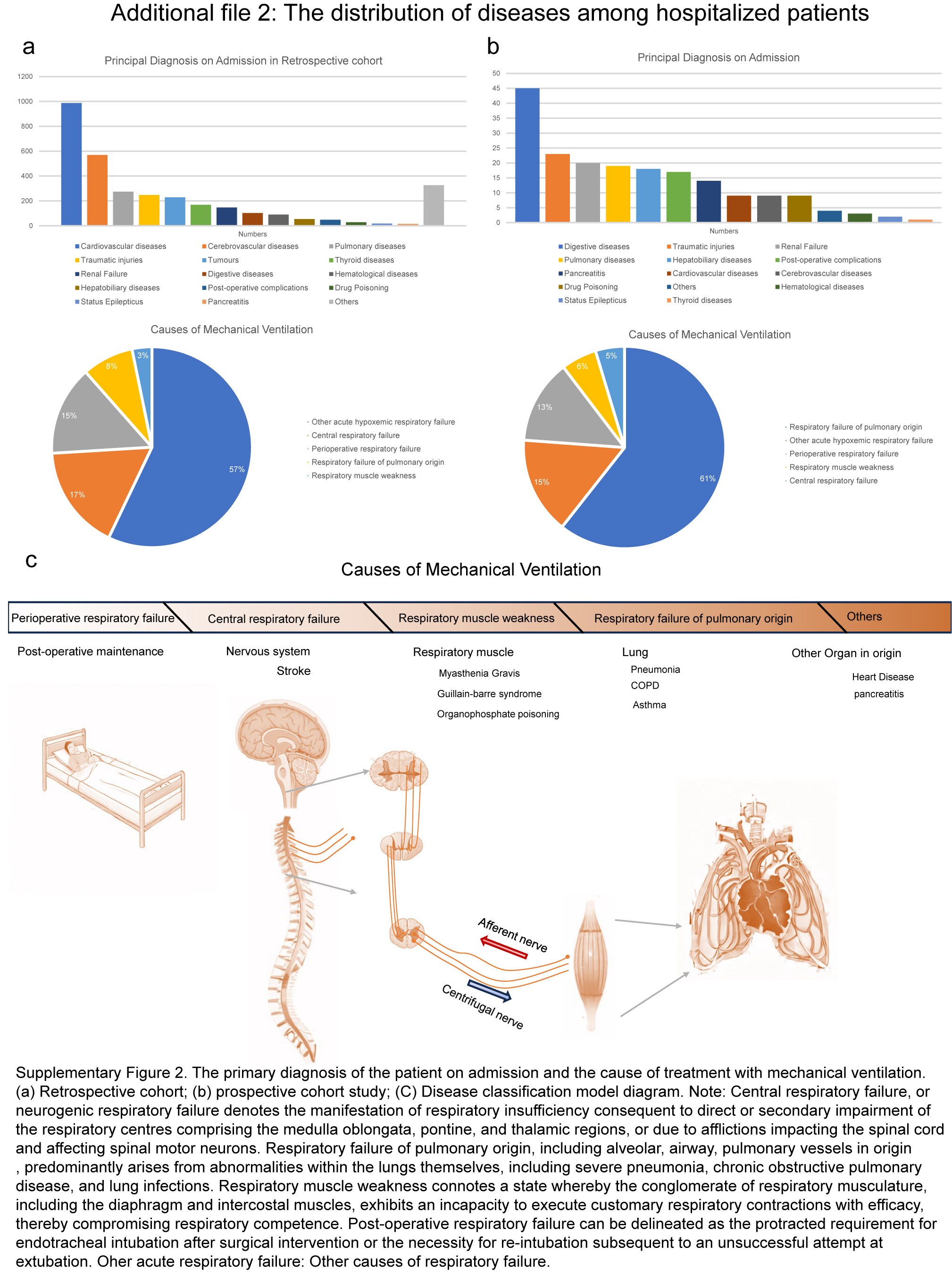

Supplement: Supplementary file 2 — Additional file 2. The distribution of diseases among hospitalized patients. [file 13054_2023_4579_MOESM2_ESM.jpg]

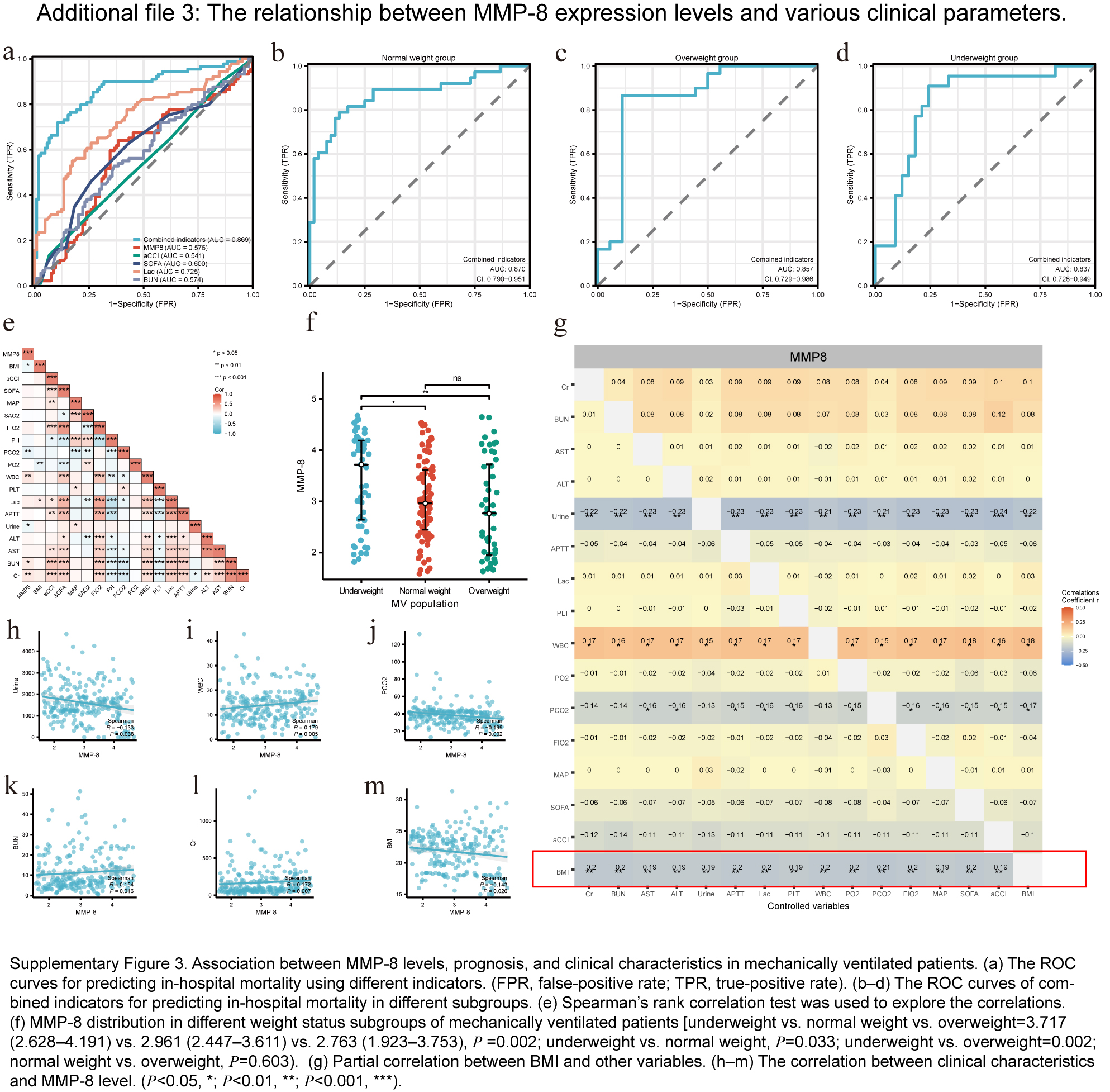

Supplement: Supplementary file 3 — Additional file 3. The relationship between MMP-8 expression levels and various clinical parameters. [file 13054_2023_4579_MOESM3_ESM.jpg]
