# Supplementary material for: Elevated MMP-8 levels, inversely associated with BMI, predict mortality in mechanically ventilated patients: an observational multicenter study
Source: Crit Care. 2023 Jul 18;27:290. doi: 10.1186/s13054-023-04579-3 (PMC10355076; doi:10.1186/s13054-023-04579-3)
Supplement: Supplementary file 4 — Additional file 4. Appendix of Supplementary Table. [file 13054_2023_4579_MOESM4_ESM.docx]

**Additional file 4: Appendix of Supplementary Table**

**Elevated MMP-8 Levels, Inversely Associated with BMI, Predict Mortality in Mechanically Ventilated Patients: An Observational Multicenter Study**

Hang Ruan^1,2^, Shu-sheng Li ^1,2^, Qin Zhang ^3*^, Xiao Ran ^1,2*^

^1^Department of Critical-care Medicine, Tongji Hospital, Tongji Medical College, Huazhong University of Science and Technology, Wuhan, 430030, China.

^2^Department of Emergency Medicine, Tongji Hospital, Tongji Medical College, Huazhong University of Science and Technology, Wuhan, China.

^3^Department of Anesthesiology, Hubei Key Laboratory of Geriatric Anesthesia and Perioperative Brain Health,

and Wuhan Clinical Research Center for Geriatric Anesthesia, Tongji Hospital, Tongji Medical College,

Huazhong University of Science and Technology, Wuhan, 430030, China.

***Corresponding authors:**

Qin Zhang

Address: 1095# Jiefang Ave, Tongji Hospital, Tongji Medical College, Huazhong University of Science and Technology, Wuhan, 430000*,* *China*. Phone: +86-15717554768; Email: qzhang8@tjh.tjmu.cn

Xiao Ran

Address: 1095# Jiefang Ave, Tongji Hospital, Tongji Medical College, Huazhong University of Science and Technology, Wuhan, 430000, China. Phone: +86-15926207366; Email: ranxiao1001@tjh.tjmu.edu.cn

Contents

[Supplementary Table 1. 3](#_Toc136741920)

[Supplementary Table 2. 4](#_Toc136741921)

[Supplementary Table 3. 5](#_Toc136741922)

[Supplementary Table 4. 6](#_Toc136741923)

[Supplementary Table 5. 7](#_Toc136741924)

[Supplementary Table 6. 8](#_Toc136741925)

# Supplementary Table 1.

Assessment of multicollinearity among the independent variables.

| Variable | VIF | SQRT VIF | Tolerance | Squared |
| --- | --- | --- | --- | --- |
| Age | 2.14 | 1.46 | 0.4665 | 0.5335 |
| aCCI | 3.34 | 1.83 | 0.2997 | 0.7003 |
| SOFA | 1.96 | 1.4 | 0.5113 | 0.4887 |
| MAP, mmHg | 1.54 | 1.24 | 0.6514 | 0.3486 |
| PCO_2_, mmHg | 1.63 | 1.28 | 0.6137 | 0.3863 |
| PLT, 10^9^/L | 1.62 | 1.27 | 0.6192 | 0.3808 |
| Lac, mmol/L | 2.4 | 1.55 | 0.4164 | 0.5836 |
| APTT, S | 1.12 | 1.06 | 0.8938 | 0.1062 |
| ALT, U/L | 2.31 | 1.52 | 0.4334 | 0.5666 |
| AST, U/L | 2.62 | 1.62 | 0.3817 | 0.6183 |
| BUN, mmol/L | 2.79 | 1.67 | 0.3578 | 0.6422 |
| Cr, µmol/L | 2.34 | 1.53 | 0.4276 | 0.5724 |
| BMI, kg/m^2^ | 1.03 | 1.02 | 0.9703 | 0.0297 |
| Mean VIF | 2.06 |  |  |  |

# Supplementary Table 2.

The unadjusted and adjusted outcomes by BMI categories in patients receiving mechanical ventilation

| Subgroup (BMI) | Unadjusted |  | Adjusted |  |
| --- | --- | --- | --- | --- |
|  | OR (95%CI) | *P*-Value | OR (95%CI) | *P*-Value |
| < 18.5 | 1.819(1.430 -2.315) | <0.001 | 1.843 (1.435 – 2.367) | <0.001 |
| 18.5 - 23.9 | Reference |  | Reference |  |
| 24.0 - 27.9 | 0.511(0.373 -0.700) | <0.001 | 0.565 (0.410 – 0.779) | <0.001 |
| 28.0 - 31.9 | 0.465 (0.254 -0.850) | 0.013 | 0.384 (0.204 – 0.723) | 0.003 |
| ≥ 32.0 | 1.070 (0.551 -2.077) | 0.841 | 0.630 (0.309 – 1.283) | 0.203 |

Note：Adjusted for age, SOFA, PaCO_2_, Lac, AST, and BUN.

# Supplementary Table 3.

Balance Checks

| IHM | Coef. | SD. | z | *P*-Value | 95% CI |
| --- | --- | --- | --- | --- | --- |
| Age | -0.091 | 0.092 | -0.99 | 0.320 | -0.271 -0.088 |
| SOFA | -0.455 | 0.442 | -1.03 | 0.303 | -1.322 -0.412 |
| PCO_2_, mmHg | 0.144 | 1.662 | 0.09 | 0.931 | -3.113 -3.401 |
| Lac, mmol/L | -0.367 | 0.234 | -1.57 | 0.116 | -0.826 -0.091 |
| AST, U/L | -27.743 | 23.515 | -1.18 | 0.238 | -73.831 -18.344 |
| BUN, mmol/L | -1.648 | 0.964 | -1.71 | 0.087 | -3.537 -0.240 |

Note: IHM, In-hospital mortality. The findings indicated that when BMI was 22.85 kg/m^2^, the mean impact of the covariates on the outcome was zero.

# Supplementary Table 4.

Expression levels of MMP-8 in different types of tumors

| Group | Test | Control | 95%CI | *P-Value* |
| --- | --- | --- | --- | --- |
| **LUAD** | Tumor | Normal | -0.068 - -0.014 | **<0.001** |
| **COAD** | Tumor | Normal | -0.173 - -0.041 | **<0.001** |
| **COADREAD** | Tumor | Normal | -0.160 - -0.042 | **<0.001** |
| **BRCA** | Tumor | Normal | -0.042 - -0.000 | **0.004** |
| ESCA | Tumor | Normal | -0.272 - 0.000 | 0.079 |
| **STES** | Tumor | Normal | -0.148 - -0.027 | **0.001** |
| KIRP | Tumor | Normal | -0.014- 0.000 | 0.404 |
| KIPAN | Tumor | Normal | -0.014 - 0.000 | 0.550 |
| **STAD** | Tumor | Normal | -0.162- -0.014 | **0.005** |
| PRAD | Tumor | Normal | -0.014 - 0.014 | 0.542 |
| UCEC | Tumor | Normal | -0.056 - 0.042 | 0.387 |
| **HNSC** | Tumor | Normal | -0.100 - -0.014 | **0.001** |
| KIRC | Tumor | Normal | -0.014 - 0.000 | 0.515 |
| **LUSC** | Tumor | Normal | -0.122 - -0.046 | **<0.001** |
| LIHC | Tumor | Normal | -0.0004 - 0.000 | 0.611 |
| THCA | Tumor | Normal | -0.014 - 0.000 | 0.215 |
| BLCA | Tumor | Normal | -0.068 - 0.014 | 0.277 |
| KICH | Tumor | Normal | -0.000 - 0.028 | 0.127 |
| CHOL | Tumor | Normal | -0.353 - 0.014 | 0.194 |

Note: Data groups with a sample size less than 3 or with a standard deviation of 0 (READ) were not included in the statistical analysis. Abbreviations: LUAD, Lung adenocarcinoma; COAD, Colon adenocarcinoma; COADREAD, Colon adenocarcinoma/Rectum adenocarcinoma Esophageal carcinoma; BRCA, Breast invasive carcinoma; ESCA, Esophageal carcinoma; STES, Stomach and Esophageal carcinoma; KIPAN, Pan-kidney cohort (KICH+KIRC+KIRP); STAD, Stomach adenocarcinoma; PRAD, Prostate adenocarcinoma; UCEC, Uterine Corpus Endometrial Carcinoma; HNSC, Head and Neck squamous cell carcinoma; KIRC, Kidney renal clear cell carcinoma; LUSC, Lung squamous cell carcinoma; LIHC, Liver hepatocellular carcinoma; THCA, Thyroid carcinoma; READ, Rectum adenocarcinoma; BLCA, Bladder Urothelial Carcinoma; KICH, Kidney Chromophobe; CHOL, Cholangiocarcinoma.

# Supplementary Table 5.

Tests for S-shaped relationships were performed by including cubic term.

| IHM | Coef. | Std. Err. | t | *P*-Value | 95% CI |
| --- | --- | --- | --- | --- | --- |
| MMP-8 | -26.665 | 7.976 | -3.34 | 0.001 | -42.297 - -11.032 |
| MMP-8^2^ | 9.193 | 2.682 | 3.43 | 0.001 | 3.937 – 14.449 |
| MMP-8^3^ | -0.994 | 0.288 | -3.44 | 0.001 | -1.559 - -0.428 |
| Cons | 23.817 | 7.549 | 3.15 | 0.002 | - 1. – 38.613 |

Note: Coef. = coefficient. Cons, constant term. Fitting Formula: y = -26.66*x + 9.19*x^2 - 0.99*x^3 + 23.82. For the cubic equation f(x) = ax^3 - bx^2 + cx + d, the vertex can also be calculated using the following steps: 1. Take the derivative of f(x) to obtain f'(x) = 3ax^2 - 2bx + c. 2. Set f'(x) to zero and solve for x, which gives $x = (2b \pm\sqrt{(4b^2- 12ac)}) / 6a$.

# Supplementary Table 6.

Comparison of the diagnostic efficacy of various indicators in predicting the IHM

| Characteristics | Cut-off values | Sensitivity (%) | Specificity (%) | AUC (95% CI) | Youden Index | *P*-Value |
| --- | --- | --- | --- | --- | --- | --- |
| Combined indicators | 0.560 | 0.719 | 0.894 | 0.869 (0.816 - 0.922) | 0.613 | <0.001 |
| MMP-8 | 3.023 | 0.640 | 0.615 | 0.576 (0.493 - 0.658) | 0.256 | 0.695 |
| aCCI | 4.500 | 0.135 | 0.933 | 0.541 (0.460 - 0.621) | 0.068 | 0.306 |
| Lac | 6.045 | 0.551 | 0.827 | 0.725 (0.652 - 0.798) | 0.377 | 0.023 |
| BUN | 11.850 | 0.528 | 0.635 | 0.574 (0.492 - 0.655) | 0.163 | 0.668 |
| SOFA | 10.500 | 0.461 | 0.740 | 0.600 (0.519 - 0.681) | 0.201 | Ref. |

Note: Combined indicators: Construction of joint predictors by MMP8, BMI, Lac, PO2, SAO2, FIO2, and BUN. DeLongs test was used for the comparison of different AUCs. SOFA score was used as reference test. The integral ROC curve values were computed for various clinical indicators to forecast in-hospital mortality and assess the diagnostic efficiency of different indicators. Findings revealed that MMP-8 level alone was only capable of providing limited prognosis for in-hospital death in mechanically ventilated patients (**Supplementary Figure 3a**). In order to improve diagnostic performance, the study put forward a composite index that combined MMP-8 levels and relevant clinical parameters, including BMI, Lac, PAO_2_, SAO_2_, FIO_2_, and BUN. The composite index showed promising potential for diagnosis, outperforming the reference SOFA score in predicting in-hospital mortality (0.869 (0.816–0.922) vs. 0.600 (0.519–0.681), *P*<0.001, Supplementary Table 4). Furthermore, the combination of indicators exhibited high diagnostic performance in different BMI groups (**Supplementary Figure 3**b–d). The ROC curve results demonstrated that MMP-8 level, combined with clinical indicators, was effective in predicting in-hospital mortality in patients (AUC (95% CI), 0.869 (0.816 - 0.922), Youden Index=0.613).
